# Predictors of adverse pathology on radical prostatectomy specimen in men initially enrolled in active surveillance for low-risk prostate cancer

**DOI:** 10.1007/s00345-020-03394-7

**Published:** 2020-07-30

**Authors:** Lars Björnebo, Henrik Olsson, Tobias Nordström, Fredrik Jäderling, Henrik Grönberg, Martin Eklund, Anna Lantz

**Affiliations:** 1 Department of Medical Epidemiology and Biostatistics, Karolinska Institutet, Solna, Sweden; 2 Department of Clinical Sciences, Danderyds Hospital, Danderyd Hospital, Danderyd, Sweden; 3 Department of Molecular Medicine and Surgery, Karolinska Institutet, Stockholm, Sweden; 4 Department of Diagnostic Radiology, Karolinska University Hospital, Stockholm, Sweden

**Keywords:** Prostate cancer, Prostate neoplasm, Active surveillance, Prostate biopsy, Magnetic resonance imaging, MRI, Prostate-specific antigen, PSA

## Abstract

**Purpose:**

To evaluate clinical variables, including magnetic resonance imaging (MRI) predictive of adverse pathology (AP) at radical prostatectomy (RP) in men initially enrolled in active surveillance (AS).

**Methods:**

A population-based cohort study of men diagnosed with low-risk prostate cancer (PCa), in Stockholm County, Sweden, during 2008–2017 enrolled in AS their intended primary treatment followed by RP. AP was defined as ISUP grade group  ≥ 3 and/or pT-stage ≥ T3. Association between clinical variables at diagnosis and time to AP was evaluated using Cox regression and multivariate logistic regression to evaluate the association between AP and clinical variables at last biopsy before RP.

**Results:**

In a cohort of 6021 patients with low-risk PCa, 3116 were selected for AS and 216 underwent RP. Follow-up was 10 years, with a median time on AS of 23 months. 37.7% of patients had AP at RP. Clinical T-stage [Hazard ratio (HR): 1.81, 95% confidence interval (CI) 1.04–3.18] and PSA (HR: 1.31, 95% CI 1.17–1.46) at diagnosis and age [Odds Ratio (OR): 1.09, 95% CI 1.02–1.18), PSA (OR: 1.22, 95% CI 1.07–1.41), and PI-RADS (OR 1.66, 95% CI 1.11–2.55)] at last re-biopsy were significantly associated with AP.

**Conclusion:**

PI-RADS score is significantly associated with AP at RP and support current guidelines recommending MRI before enrollment in AS. Furthermore, age, cT-stage, and PSA are significantly associated with AP.

**Electronic supplementary material:**

The online version of this article (10.1007/s00345-020-03394-7) contains supplementary material, which is available to authorized users.

## Introduction

Active surveillance (AS) is standard of care for men with low-risk prostate cancer (PCa) to avoid overtreatment of indolent cancers. Most, 80–90%, men who die within 10–15 years after diagnosis of low-risk PCa will die from other causes than PCa [1, 2]. European Association of Urology (EAU) recommend that AS should be offered to patients with treatable low-risk PCa [3, 4]. The inclusion criteria for AS aim to maximize the number of patients eligible for AS to avoid overtreatment while simultaneously minimizing the risk of missing the window of curability. There are multiple different inclusion criteria for AS [5–7]. Among the most widely used inclusion criteria are the Prostate Cancer Research International Active Surveillance (PRIAS) criteria, where men with T1/T2 PCa, PSA ≤ 10 ng/ml, PSA density < 0.2 ng/ml per milliliter, one or two positive biopsy cores, and biopsy-GG (ISUP grade group) ≤ 1 are eligible [8]. Additionally, current EAU guidelines recommend AS for favorable biopsy-GG 2 PCa [3]. A 10-year follow-up study of men on AS, evaluating predictors for unfavorable outcome at RP, found that biopsy-GG upgrading to > 1 and T3 should be the only triggers for an immediate switch to treatment, and that an increase in PSA and/or more than two positive cores should instigate further investigation rather than immediate treatment [9].

Some prior studies exist in regard to predicting upgrading at RP after initial AS. Reese et al. evaluated 130 patients initially on AS who underwent RP and concluded that disease reclassification during AS was the only significant factor associated with adverse pathology (AP) at RP [10]. Other studies have looked at predictors of AP in patient populations eligible, but not enrolled in AS [11–13]. Only the PRIAS study included a statistical analysis of which variables observed during AS could predict AP at RP [9]. The aim of this study was to evaluate predictors for AP in a large population-based cohort before and during AS on prostate specimen after RP. To our knowledge, this is the largest cohort of patients undergoing RP after the initial enrollment in AS, including data on MRI, which has been analyzed regarding this research question.

## Patients and methods

### Study design

This is a retrospective, cross-sectional study of men diagnosed with low-risk PCa who underwent RP after being enrolled in AS in the Stockholm County. Patients were diagnosed and operated between January 2008 and December 2017. Data on tumor characteristics and primary treatment were sourced from the National Prostate Cancer Register (NPCR), which includes complete data on all diagnosed PCa in Sweden [14]. PSA and biopsy data were retrieved from all laboratories in Stockholm County. Out of 6021 patients between the ages of 40 and 75 years diagnosed with low-risk and very-low-risk PCa (biopsy-GG = 1, PSA < 10, pT-stage ≤ 2), 3116 patients enrolled in AS were identified (Fig. S1). Swedish guidelines for AS patients, applicable during the study period, suggest repeat biopsies within 2–6 months of diagnostic biopsy followed by biopsies every 2–3 years, repeat PSA tests every 3–4 months during the first 2 years and every 6 months after that [4]. The patients in this study, after some time on AS, finally underwent RP as curative treatment for their PCa. Data on biopsy-GG, pT-stage, mm cancer, number of biopsy cores, and number of positive cores were collected from diagnostic and surveillance biopsies. In a journal review, data on MRI and pathology report data from RP specimens were collected, which included data on RP-GG, pT-stage, and surgical margins. MRI scans were reported by clinical board-certified radiologists and performed at both academic and non-academic centers. Data on MRI included PI-RADS score, extracapsular extension, and number of lesions. Not all patients underwent an MRI, since this was not in the guidelines at the time of the study period.

### Statistical analysis

The primary outcome was upgrading to AP at RP. AP was defined as either RP-GG ≥ 3 and/or pT-stage ≥ T3 disease at RP. Two separate analyses were performed, from diagnosis of PCa as well as from the last surveillance biopsy before RP.

In the analysis from diagnosis of PCa to time to AP at RP, hazard ratios (HR) were calculated using Cox proportional hazard models. If AP was not observed at time of RP, right-censoring was applied. Previously well-established clinical variables were selected for univariate analysis from diagnosis to RP, which included age at diagnosis, family history of PCa, clinical T-stage, PSA, PSA density, prostate volume, ratio of positive biopsy cores, and mm cancer. The non-correlated relevant variables were selected for a multivariate Cox proportional hazard model to calculate P-values and HRs with 95% confidence intervals (CI).

In the separate analysis of AP from last surveillance biopsy in AS to RP, patients with at least one re-biopsy that had been performed within 12 months of the RP were selected. The last available data on the variables were selected. Both univariate and multivariate analyses were performed. Multivariate logistic regression was used to calculate *P* values and Odds Ratios (OR) with 95% CIs. In addition to the variables mentioned above, this analysis included time on AS, time since last biopsy, PI-RADS score, and number of lesions on MRI. A two-sided *P* value of < 0.05 was considered significant for all analyses. Statistical analysis was performed using R version 3.6.1 [15].

## Results

A total of 6021 men were identified with low- and very-low-risk PCa between January 2008 and December 2017. 3116 (51.8%) were enrolled in AS their primary treatment, and out of these men, 216 (6.9%) underwent RP with a median time on AS of 23 (5–109, Q1–Q3) months. Complete RP pathology data were available for 212 out of 216 patients. Table 1 shows the patient and disease characteristics at diagnosis for all patients with low- and very-low-risk PCa, patients enrolled in in AS, and patients who underwent RP.

**Table 1:** Patient characteristics

	Overall (*n* = 6021)	AS cohort (*n* = 3116)	*P* value^a^	RP cohort (*n* = 212)	*P* value^b^
Age at diagnosis			< 0.0001		0.07
< 60	1688 (28.2%)	725 (23%)		56 (26.4%)	
60–69	3387 (56%)	1869 (56%)		129 (60.8%)	
≥ 70	946 (16%)	522 (17%)		27 (12.7%)	
Family history of PCa*			0.02		0.02
No	4989 (83%)	2639 (85%)		165 (77.8%)	
Yes	1032 (17%)	555 (19%)		47 (22.2%)	
cT-stage			< 0.0001		0.05
T1	5020 (83%)	2761 (89%)		178 (84%)	
T2	914 (15%)	314 (10%)		32 (15.1%)	
Missing	87 (1.4%)	41 (1.3%)		2 (1%)	
PSA (ng/ml)			0.004		0.005
0–3	781 (12.5%)	451 (14.7%)		24 (11.3%)	
3–5	2381 (40%)	1252 (40%)		72 (34%)	
5–10	2859 (48%)	1413 (45%)		116 (54.7%)	
≥ 10	0 (0%)	0 (0%)		0 (0%)	
PSA density (ng/ml/ml)			< 0.0001		< 0.0001
< 0.15	3618 (60%)	2094 (67.2%)		113 (53.3%)	
≥ 0.15	2120 (35.2%)	894 (28.7%)		96 (45.3%)	
Missing	283 (4.7%)	128 (4.1%)		3 (1.4%)	
Prostate volume (ml)			< 0.0001		< 0.0001
Median (Q1–Q3)	37 (28–50)	40 (30–52)		33 (27–43)	
Missing	283	128		3	

Data are presented as median and Q1–Q3 for continuous variables and as numbers and percentages for ordinal and categorical variables

*RP* radical prostatectomy, *AS* active surveillance

*Any first-degree family member diagnosed with PCa

^a^AS cohort compared with overall cohort

^b^RP cohort compared with AS cohort

Table 2 shows the changing disease characteristics from diagnosis, via last biopsy, to RP. 37.7% of patients had AP at RP. 26.5% of patients had T3 disease at RP. 70.7% of patients had GG ≥ 2 at last surveillance biopsy compared to 83.5% at RP (Fig. S2). 76% of patients were reclassified, based on PSA ≥ 10 and/or biopsy-GG ≥ 2, before RP. 42% of the reclassified men had AP at RP compared to 24% of men who were not reclassified. 70.8% of patients were upgraded to biopsy-GG ≥ 2 and 23.1% presented with an elevated PSA > 10 before RP. 23.6% of the patients were not upgraded preoperatively and underwent RP due to other causes. Most patients (76.9%) had an MRI during surveillance, and 63.8% of these patients had an associated PI-RADS score. MRI and PI-RADS score were introduced into clinical practice during the study period, with 57% and 90.5% of patients having an MRI with 2015 and 2017 as their year of RP, respectively (Fig. S3).

**Table 2:** Disease characteristics at diagnosis, last biopsy, and radical prostatectomy

	Diagnostic biopsy (*n* = 212)	Last biopsy before RP (*n* = 212)	RP (*n* = 212)
Age at diagnosis/age at RP			
< 60	56 (26.4%)		39 (18.4%)
60–69	129 (60.8%)		118 (55.7%)
70 +	27 (12.7%)		55 (25.9%)
cT/pT-stage			
T1	180 (84.9%)	156 (73.6%)	0 (0%)
T2	32 (15.1%)	56 (26.4%)	155 (73.1%)
T3a	0 (0%)	0 (0%)	51 (24.1%)
T3b	0 (0%)	0 (0%)	5 (2.4%)
Missing			1 (0.5%)
PSA (ng/ml)			
0–3	24 (11.3%)	14 (6.6%)	
3–5	72 (34.0%)	51 (24.1%)	
5–10	116 (54.7%)	98 (46.2%)	
10 +	0 (0%)	49 (23.1%)	
PSA density (ng/ml/ml)			
< 0.15	113 (53.3%)	78 (36.8%)	
≥ 0.15	96 (45.3%)	134 (63.2%)	
Missing	3 (1.4%)	0 (0%)	
ISUP grade group			
1	212 (100.0%)	62 (29.2%)	35 (16.5%)
2	0 (0%)	110 (51.9%)	126 (59.4%)
3	0 (0%)	31 (14.6%)	36 (17.0%)
≥ 4	0 (0%)	9 (4.2%)	15 (7.1%)
Surgical margins			
Negative			144 (67.9%)
Positive			66 (31.1%)
Missing			2 (.9%)
Adverse pathology (≥ T3 and/or ISUP ≥ 3)			
No			131 (61.8%)
Yes			80 (37.7%)
Missing			1 (.5%)
Time since last biopsy before RP (months)			
Median (Q1–Q3)			4 (2–7)
Number of re-biopsies			
0		14 (6.6%)	
1		112 (52.8%)	
2		55 (25.9%)	
3 +		31 (14.6%)	
Ratio of pos cores (%)			
Median (Q1–Q3)	17 (10–25)	33 (25–58)	
mm cancer (mm)			
Median (Q1–Q3)	3 (1.5–6)	12 (6–20)	
Missing	3	0	
Time on AS (months)			
Median (min–max)		23 (5–109)	
MRI			
Yes		163 (76.9%)	
No		49 (23.1%)	
PIRADS			
≤ 2		22 (13.5%)	
3		19 (11.7%)	
4		38 (23.3%)	
5		45 (27.6%)	
Missing		39 (23.9%)	
Number of lesions			
0		22 (13.5%)	
1		88 (54.0%)	
2		44 (27.0%)	
3		9 (5.5%)	
Targeted biopsy			
Yes		51 (31.3%)	
No		112 (68.7%)	

*RP* radical prostatectomy, *AS* active surveillance

Among men with a highest PI-RADS-score of ≤ 2, 3, 4, and 5 at the latest MRI, the proportion having AP at radical prostatectomy was 15%, 16%, 39%, and 57%, respectively. Among men with a PSA density of ≥ 0.15, 45% had AP at RP compared to 26% of men with a PSA density of < 0.15.

**Fig. 1:**
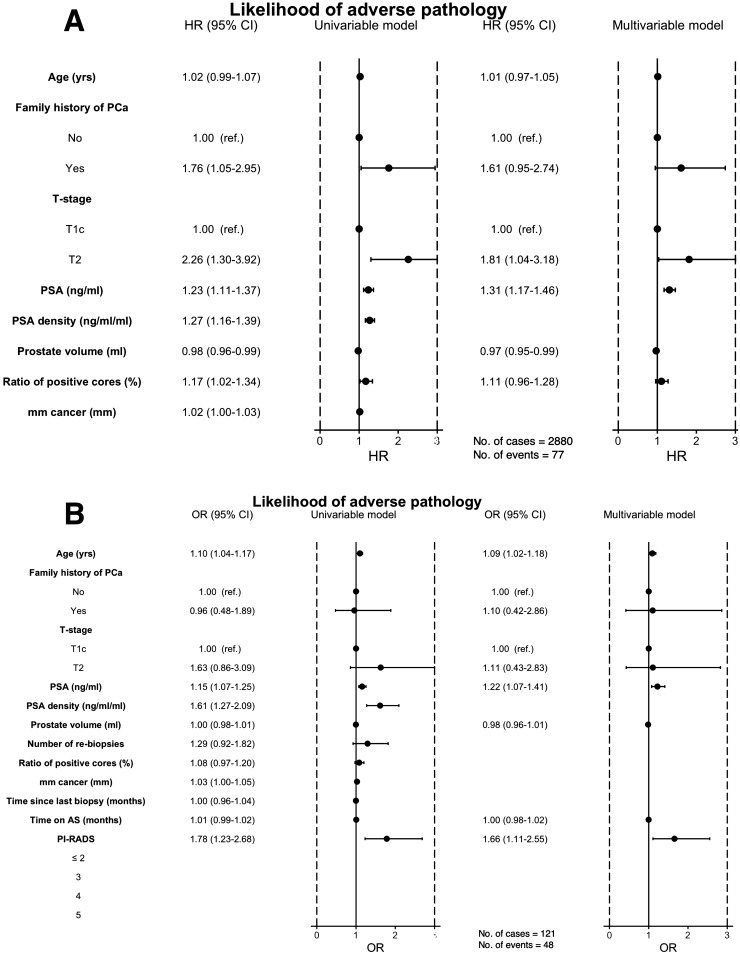
Association of patient and tumor characteristics with adverse pathology: univariate and multivariate analyses. From diagnosis to radical prostatectomy (**a**). From biopsy to radical prostatectomy (**b**). PSA increase of 1 unit. PSA density and ratio of positive cores increase of 0.1 units. OR for PI-RADS is based on one-unit increase in PI-RADS PSA increase of 1 unit. *AS* active surveillance, *PCa* prostate cancer

From diagnosis of PCa, univariate analysis showed that significant predictors of AP at RP were family history of PCa, cT-stage, PSA, PSA density, ratio of positive biopsy cores, and mm cancer at biopsy. Multivariate analysis showed that cT-stage (T2) (HR: 1.81, 95% CI 1.04–3.18), PSA (HR: 1.31, 95% CI 1.17–1.46), and prostate volume (HR: 0.97, 95% CI 0.95–0.99) were significantly associated with AP (Fig. 1a). Age, family history of PCa, prostate volume, and ratio of positive cores at diagnosis were not significantly associated with AP in the multivariate analysis.

In a separate analysis of AP at RP with variables from the last surveillance, significant predictors of AP on univariate analysis were increasing age, PSA, PSA density, mm cancer at biopsy, and PI-RADS (Fig. 1b). Age (OR: 1.09, 95% CI 1.02–1.18), PSA (OR: 1.22, 95% CI 1.07–1.41), and PI-RADS (OR: 1.66, 95% CI 1.11–2.55) were significantly associated with AP in multivariate analysis. Time on AS, family history of PCa, cT-stage, and prostate volume were not associated with an increased risk of AP. The multivariate model included only the 121 patients with data on PI-RADS score. In a sensitivity analysis, a separate multivariate analysis of all 212 patients, excluding PI-RADS, showed the same variables as being significant.

## Discussion

In this large population-based AS cohort, cT-stage and PSA at diagnosis of PCa were significantly associated with AP at RP. At the last surveillance biopsy before RP, age, PSA, and PI-RADS score were significant predictors of AP at RP. Importantly, no association between time on AS and AP was found. Our results emphasize the importance and difficulty in selecting the right patients for AS and suggest that improvements in diagnostic precision before enrollment and during AS, with PSA, digital rectal exam, confirmatory biopsy, and MRI, would aid the decision-making.

### Predicting adverse pathology at radical prostatectomy

Significant predictors of AP on RP varied slightly between analysis from diagnosis and from last re-biopsy. In both analyses, PSA was a predictor of AP, and at diagnosis, cT-stage was significantly associated with AP. Other studies have linked PSA and PSA density to AP at RP [10, 11, 13]. However, most of these studies include patients that could potentially be enrolled in AS and few of them are actually based on patients enrolled in AS. Older age and its link to AP at RP, shown in other studies, were confirmed in this study [16, 17]. Some biopsy variables, percent positive biopsy cores, and total mm cancer, previously shown to have a significant association with AP, did not show an association in this study, which possibly could be explained by differences in biopsy sampling in a patient population that is not enrolled in AS [13]. Additionally, 31.3% of the patients in this study underwent a targeted biopsy which could affect mm cancer and number of positive cores detected. In the largest study (PRIAS) of AP at RP after initial AS, AP was only significantly associated with biopsy-GG > 1 on last biopsy [9]. However, PSA density, MRI, and time on AS were not included in the PRIAS study and more than 50% of the patients discontinued AS due to PSA doubling time and/or > 2 positive biopsy cores. Comparing the results at RP in our study to the PRIAS study showed that favorable pathology (RP-GG 1 and pT2) was observed in 17% and 34%, respectively, which could indicate overtreatment in the PRIAS study.

### MRI and active surveillance

Our results show that PI-RADS score on MRI was significantly associated with AP at RP. This is in line with today’s AS guidelines recommending a prostate MRI before AS enrollment for an accurate disease staging and a better informed initial decision of AS [3, 4, 18]. Previous evidence has shown that in men on AS, a positive MRI is more likely to be associated with upgrading to biopsy-GG ≥ 2 than a negative MRI [19, 20]. A randomized 2-year post-biopsy follow-up study (ASIST) showed that MRI with systematic and targeted biopsies resulted in 50% fewer AS failures and upgrading of PCa compared to only systematic biopsies (SBx) [21]. Regarding MRI during surveillance, there are a few prospective studies evaluating MRI-based AS protocols, all indicating that targeted biopsies (TBx) increases the detection of clinically significant PCa (csPCa) compared to SBx alone [22–25]. In addition to increasing the detection rate of csPCa, MRI-based AS protocols also aim to reduce invasive surveillance through fewer biopsies. However, several studies have shown that the risk for MRI negative tumors in men on AS is not negligible [22, 26]. In our study, 14 out of 212 patients did not have a confirmatory re-biopsy before RP, most of whom had a PI-RADS score of 4 or 5. This could reflect patient anxiety or lack of compliance to protocols by the treating physician [27]. Previous evidence shows lack of compliance in surveillance biopsies during AS [28]. Our group reported that only 42% in this AS cohort were re-biopsied within the first year, despite guideline recommendations [29]. Compliance to AS protocol also decreases over time [29, 30]. Likely, adherence would increase if future AS protocols were optimized towards less invasive surveillance, i.e., by implementing mpMRI into AS regimens.

### Strengths and limitations

This is one of the largest population-based AS cohorts including data on disease progression and treatment. The Stockholm PSA and Biopsy Register contains data on clinical variables, PSA values, and re-biopsies which give important information on predictors of cancer progression. Furthermore, our study includes data from several population-based registers with near-complete coverage. Apart from the retrospective design, a limitation of our study is the selected cohort of AS patients undergoing surgery where 70.8% of patients were upgraded to biopsy-GG > 1 before RP. This might be explained by a primary misclassification of PCa at diagnosis and highlights the importance of optimized diagnostics before enrollment into AS. Furthermore, as MRI was not in the guidelines at the time of the study, only 76.9% had an MRI before or during AS and only the last available MRI was included. Data on the purpose of the MRI, part of AS protocol before inclusion or staging before surgery, were also not available for this study. Additionally, targeted biopsies performed during AS were a combination of cognitive and fusion biopsies, which could affect biopsy variables.

## Conclusions

In this population-based large AS cohort, the conversion rate to RP was low during the first years of surveillance. PI-RADS score on MRI is significantly associated with AP at RP which supports the current guidelines that recommend the use of MRI before enrollment in AS to reduce risk of misclassification at diagnosis of PCa. Age, PSA, and clinical stage at diagnosis predict AP at RP, suggesting that these variables are still valid for selection of appropriate AS patients. Our findings may aid in the decision-making for selection and treatment decisions in men on AS.

## Electronic supplementary material

Below is the link to the electronic supplementary material.

Supplementary file1 Fig. S1 Flowchart of the study population (GG = ISUP grade group. NPCR = National Prostate Cancer Register) (TIFF 14826 kb)

Supplementary file2 Fig. S2 ISUP grade group (GG) at diagnosis, last biopsy, and radical prostatectomy (RP) (PNG 616 kb)

Supplementary file3 Fig. S3 Uptake of MRI and PI-RADS across year of radical prostatectomy (TIFF 12228 kb)

## Data Availability

This manuscript has associated data which will not be deposited.

## Code availability

Available upon request.

## References

[1] Johansson JE, Andrén O, Andersson SO, Dickman PW, Holmberg L, Magnuson A (2004). Natural history of early, localized prostate cancer. J Am Med Assoc.

[2] Kessler B, Albertsen P (2003). The natural history of prostate cancer. Urol Clin N Am.

[3] Mottet N, Bellmunt J, Bolla M, Briers E, Cumberbatch MG, De Santis M (2017). EAU-ESTRO-SIOG guidelines on prostate cancer. Part 1: screening, diagnosis, and local treatment with curative intent. Eur Urol.

[4] Andrén O, Thellenberg Carlsson C, Styrke J, Johansson E, Åström L, Carlsson S et al. (2019) Nationellt vårdprogram prostatacancer

[5] Klotz L, Vesprini D, Sethukavalan P, Jethava V, Zhang L, Jain S (2015). Long-term follow-up of a large active surveillance cohort of patients with prostate cancer. J Clin Oncol.

[6] Dall’Era MA, Konety BR, Cowan JE, Shinohara K, Stauf F, Cooperberg MR (2008). Active surveillance for the management of prostate cancer in a contemporary cohort. Cancer.

[7] Tosoian JJ, Mamawala M, Epstein JI, Landis P, Wolf S, Trock BJ (2015). Intermediate and longer-term outcomes from a prospective active-surveillance program for favorable-risk prostate cancer. J Clin Oncol.

[8] Bul M, Zhu X, Valdagni R, Pickles T, Kakehi Y, Rannikko A (2013). Active surveillance for low-risk prostate cancer worldwide: the PRIAS study. Eur Urol.

[9] Bokhorst LP, Valdagni R, Rannikko A, Kakehi Y, Pickles T, Bangma CH (2016). A Decade of active surveillance in the PRIAS study: an update and evaluation of the criteria used to recommend a switch to active treatment. Eur Urol.

[10] Reese AC, Feng Z, Landis P, Trock BJ, Epstein JI, Carter HB (2015). Predictors of adverse pathology in men undergoing radical prostatectomy following initial active surveillance. Urology.

[11] De Cobelli O, Terracciano D, Tagliabue E, Raimondi S, Bottero D, Cioffi A (2015). Predicting pathological features at radical prostatectomy in patients with prostate cancer eligible for active surveillance by multiparametric magnetic resonance imaging. PLoS ONE.

[12] Kaye DR, Qi J, Morgan TM, Linsell S, Ginsburg KB, Lane BR (2019). Pathological upgrading at radical prostatectomy for patients with Grade Group 1 prostate cancer: implications of confirmatory testing for patients considering active surveillance. BJU Int.

[13] Vellekoop A, Loeb S, Folkvaljon Y, Stattin P (2014). Population based study of predictors of adverse pathology among candidates for active surveillance with Gleason 6 prostate cancer. J Urol.

[14] Van Hemelrijck M, Wigertz A, Sandin F, Garmo H, Hellström K, Fransson P (2013). Cohort profile: the national prostate cancer register of sweden and prostate cancer data base Sweden 2.0. Int J Epidemiol.

[15] R Development Core Team 3.0.1 (2013) A language and environment for statistical computing. R Found Stat Comput 2. https://www.R-project.org

[16] Wong LM, Neal DE, Johnston RB, Shah N, Sharma N, Warren AY (2012). International multicentre study examining selection criteria for active surveillance in men undergoing radical prostatectomy. Br J Cancer.

[17] Gershman B, Dahl DM, Olumi AF, Young RH, McDougal WS, Wu CL (2013). Smaller prostate gland size and older age predict Gleason score upgrading. Urol Oncol Semin Orig Investig.

[18] Mohler JL, Antonarakis ES, Armstrong AJ, D’Amico AV, Davis BJ, Dorff T (2019). Prostate cancer, version 2.2019, NCCN clinical practice guidelines in oncology. J Natl Compr Cancer Netw.

[19] Jayadevan R, Felker ER, Kwan L, Barsa DE, Zhang H, Sisk AE (2019). Magnetic resonance imaging-guided confirmatory biopsy for initiating active surveillance of prostate cancer. JAMA Netw Open.

[20] Schoots IG, Petrides N, Giganti F, Bokhorst LP, Rannikko A, Klotz L (2015). Magnetic resonance imaging in active surveillance of prostate cancer: a systematic review. Eur Urol.

[21] Klotz L, Pond G, Loblaw A, Sugar L, Moussa M, Berman D (2020). Randomized study of systematic biopsy versus magnetic resonance imaging and targeted and systematic biopsy in men on active surveillance (ASIST): 2-year postbiopsy follow-up. Eur Urol.

[22] Amin A, Scheltema MJ, Shnier R, Blazevski A, Moses D, Cusick T (2019). The magnetic resonance imaging in active surveillance “MRIAS” trial: use of baseline multiparametric magnetic resonance imaging and saturation biopsy to reduce the frequency of surveillance prostate biopsies. J Urol.

[23] Hamoen EHJ, Hoeks CMA, Somford DM, van Oort IM, Vergunst H, Oddens JR (2019). Value of serial multiparametric magnetic resonance imaging and magnetic resonance imaging–guided biopsies in men with low-risk prostate cancer on active surveillance after 1 yr follow-up. Eur Urol Focus.

[24] Gallagher KM, Christopher E, Cameron AJ, Little S, Innes A, Davis G (2019). Four-year outcomes from a multiparametric magnetic resonance imaging (MRI)-based active surveillance programme: PSA dynamics and serial MRI scans allow omission of protocol biopsies. BJU Int.

[25] Elkjær MC, Andersen MH, Høyer S, Pedersen BG, Borre M (2018). Multi-parametric magnetic resonance imaging monitoring patients in active surveillance for prostate cancer: a prospective cohort study. Scand J Urol.

[26] Llorente C (2020). Re: role of changes in magnetic resonance imaging or clinical stage in evaluation of disease progression for men with prostate cancer on active surveillance. Eur Urol.

[27] Ehdaie B, Assel M, Benfante N, Malhotra D, Vickers A (2017). A systematic approach to discussing active surveillance with patients with low-risk prostate cancer. Eur Urol.

[28] Loeb S, Walter D, Curnyn C, Gold HT, Lepor H, Makarov DV (2016). How active is active surveillance? Intensity of followup during active surveillance for prostate cancer in the United States. J Urol.

[29] Olsson H, Nordström T, Clements M, Grönberg H, Lantz AW, Eklund M (2019). Intensity of active surveillance and transition to treatment in men with low-risk prostate cancer. Eur Urol Oncol.

[30] Kalapara AA, Verbeek JFM, Nieboer D, Fahey M, Gnanapragasam V, Van Hemelrijck M (2020). Adherence to active surveillance protocols for low-risk prostate cancer: results of the movember foundation’s global action plan prostate cancer active surveillance initiative. Eur Urol Oncol.

