# Supplementary figures and images for: Predictors of adverse pathology on radical prostatectomy specimen in men initially enrolled in active surveillance for low-risk prostate cancer
Source: World J Urol. 2020 Jul 30;39(6):1797–804. doi: 10.1007/s00345-020-03394-7 (PMC8217019; doi:10.1007/s00345-020-03394-7)

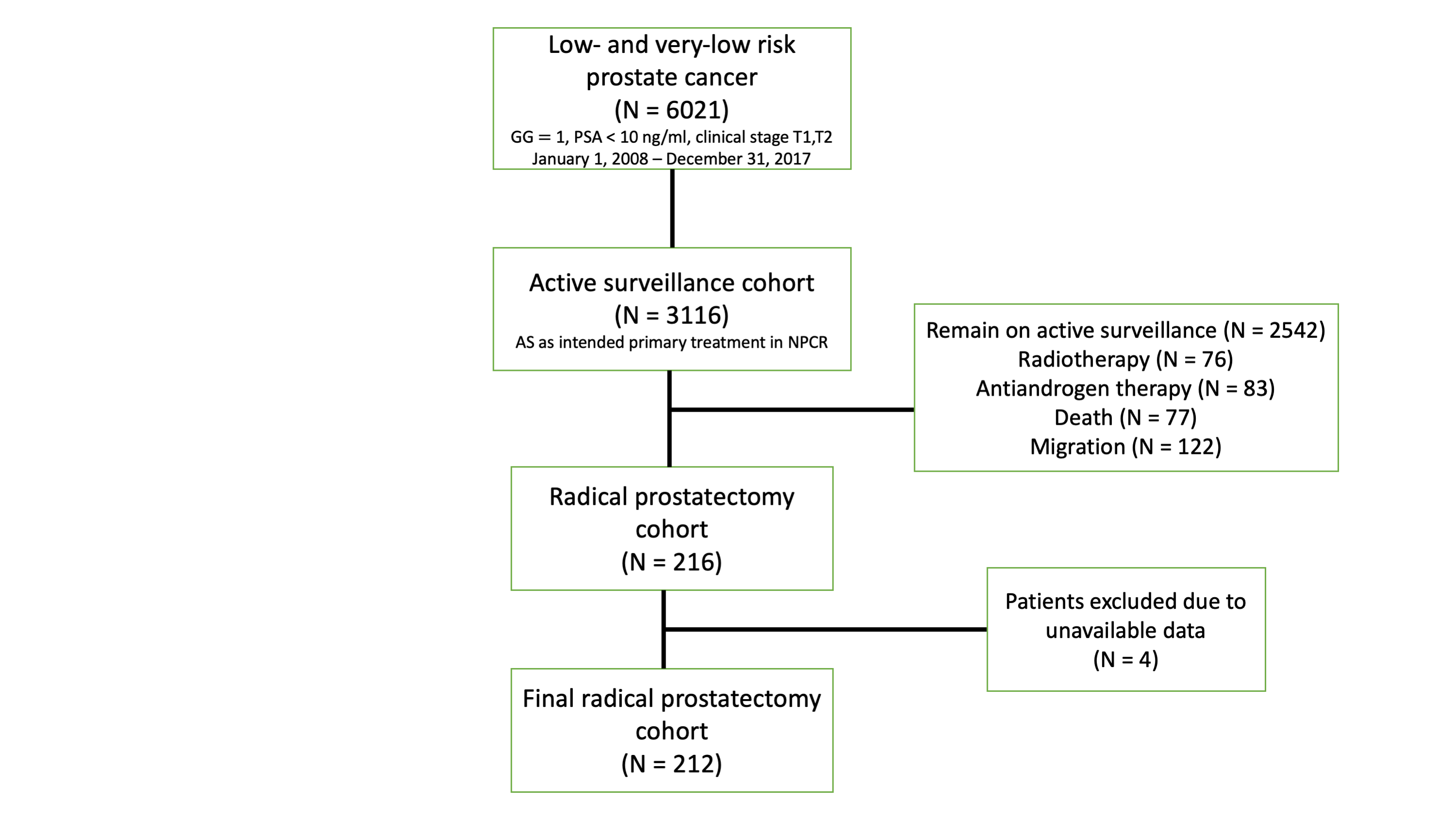

Supplement: Supplementary file 1 — Supplementary file1 Fig. S1 Flowchart of the study population (GG = ISUP grade group. NPCR = National Prostate Cancer Register) (TIFF 14826 kb) [file 345_2020_3394_MOESM1_ESM.tiff]

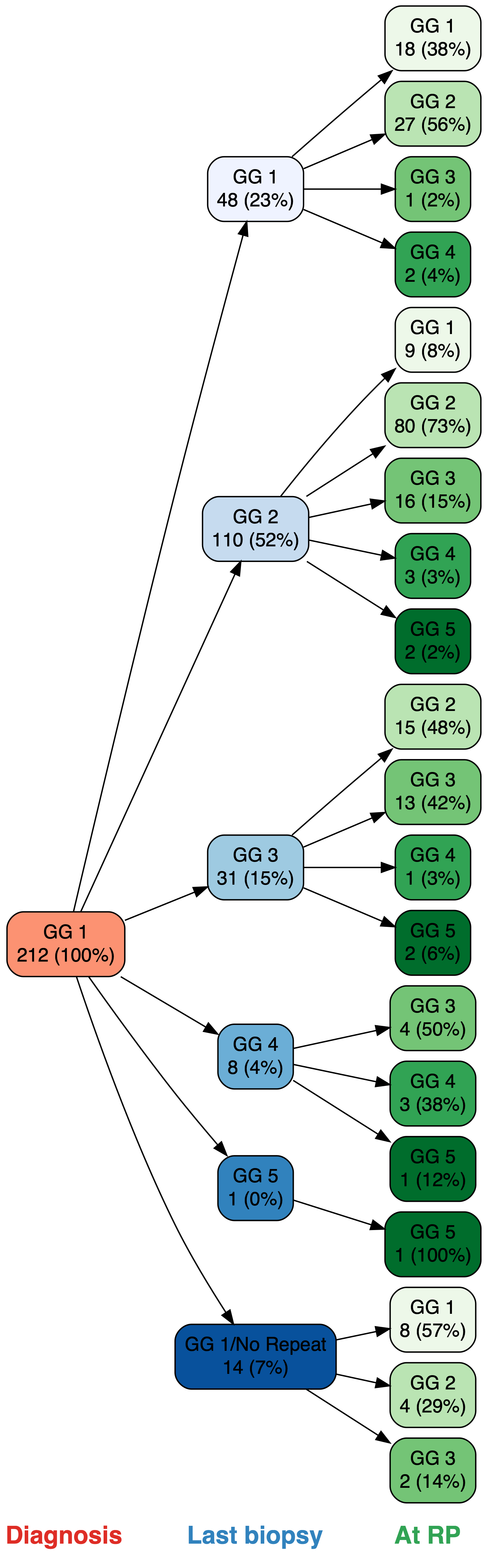

Supplement: Supplementary file 2 — Supplementary file2 Fig. S2 ISUP grade group (GG) at diagnosis, last biopsy, and radical prostatectomy (RP) (PNG 616 kb) [file 345_2020_3394_MOESM2_ESM.png]

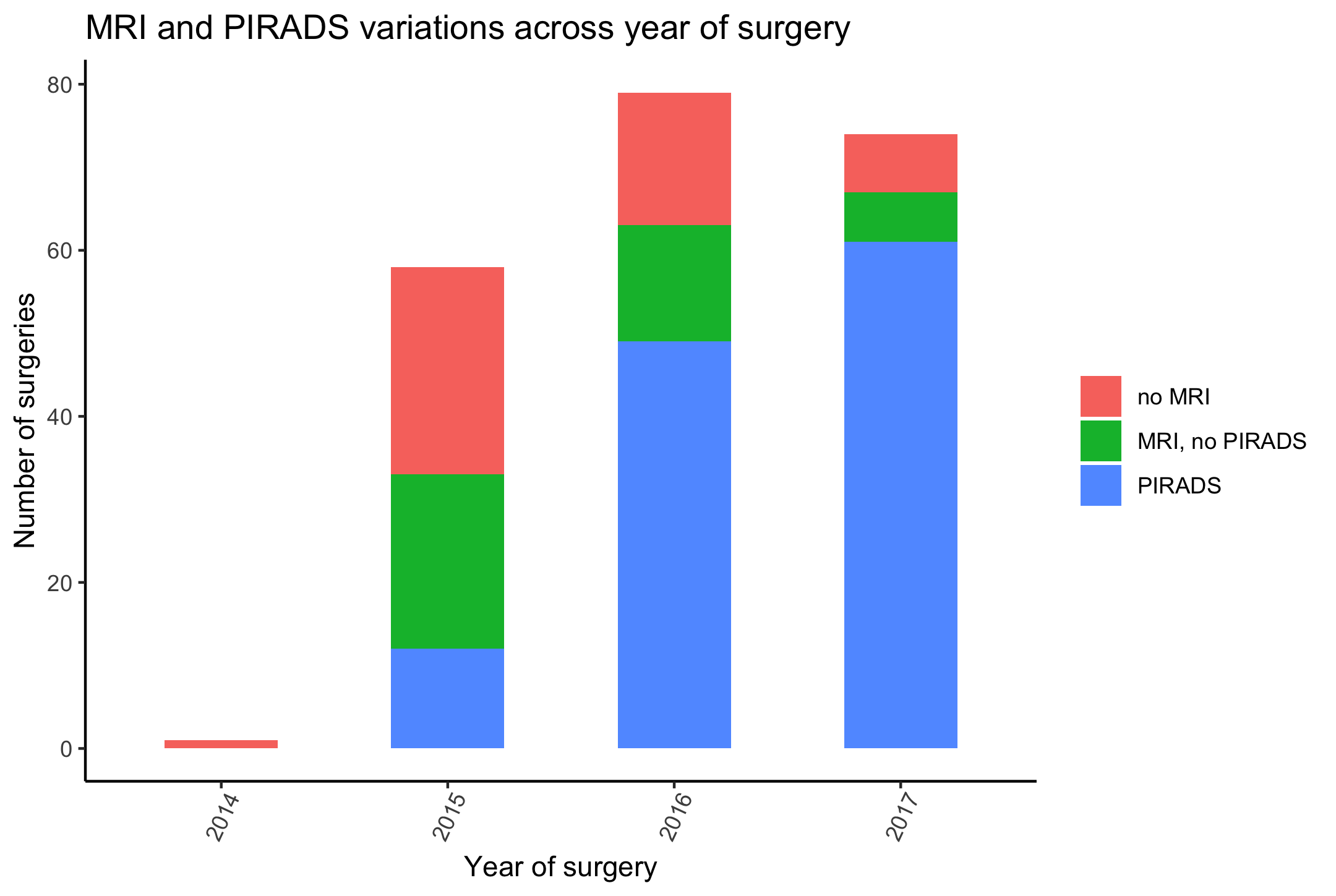

Supplement: Supplementary file 3 — Supplementary file3 Fig. S3 Uptake of MRI and PI-RADS across year of radical prostatectomy (TIFF 12228 kb) [file 345_2020_3394_MOESM3_ESM.tiff]
